# Diagnostic significance of magnetic resonance imaging in distinguishing temporomandibular disorders: a retrospective chart review

**DOI:** 10.1186/s12903-021-01826-3

**Published:** 2021-09-28

**Authors:** Nuonuo Cong, Ning Wang, Shengyuan Huang, Taiqi Cheng, Xing Yan

**Affiliations:** Department of Stomatology, Beijing Friendship Hospital, Capital Medical University, No. 95, Yongan Road, Xicheng District, Beijing, 100050 China

**Keywords:** Temporomandibular disorder, Diagnosis, Osteoarthrosis, Magnetic Resonance Imaging

## Abstract

**Background:**

This study was to evaluate the diagnostic significance of Magnetic Resonance Imaging (MRI) in distinguishing temporomandibular disorders (TMD).

**Methods:**

A total of 684 patients with TMD were included in the study. Diagnosis for TMD was conducted according to the international criteria. Two professional radiologists were selected for professional training, and the Kappa values were compared for the diagnosis results to determine the consistency of the diagnosis. Then MRI images of these 684 patients were analyzed and the diagnosis results were obtained.

**Results:**

MRI can be used for the diagnosis of TMD. There were significantly more females (518 cases) than males (166 cases) with TMD; Disc displacement with/without reduction is more common in the youth group, with the majority aged 20–30 years. The highest incidence of temporomandibular joint osteoarthrosis is in the 60-year-old age group, followed by the 70-year-old age group.

**Conclusions:**

Bilateral temporomandibular joint MRI can clearly show their changes; there are significantly more female with TMD than male; osteoarthritis has a significant correlation with age.

## Background

Temporomandibular disorders (TMD) is a common disease in the oral and maxillofacial region, and the pathogenesis is not fully understood [1]. Temporomandibular joint (TMJ) is a bilateral linkage joint consisting of the upper temporal fossa and joint nodule (both of which are also known as temporal articular surface), the lower mandibular condyle, the articular disc between the two, and the joint capsule and the ligaments inside and outside the capsule. Early functional disorders of TMD are often preclinical, self-limiting and self-healing. Many patients are stable at a certain stage without continuing to develop. For example, some patients who developed to the stage of mid-term structural disorder could still return to the early stage of the lesion after appropriate treatment. The main clinical symptoms of TMD are abnormal mandibular movement, pain, popping and/or snapping noise during both opening and closing movement. Clinical classification of TMD is divided into four categories: temporomandibular joint disorders, masticatory muscle disorders, headache and associated structures [2].

TMD are mainly diagnosed according to the clinical manifestations and signs of patients, and also need the assistance of imaging examination. X-ray and CT scan are commonly used auxiliary imaging examinations for the diagnosis of TMD. However, it is difficult to see the changes of soft tissue around the TMJ and intra-articular effusion on X-ray and CT images. With the development of imaging technology, the application of magnetic resonance imaging (MRI) in the diagnosis of temporomandibular disorders (TMD) has become more and more mature. In recent years, bilateral temporomandibular joint MRI has been gradually applied in clinical practice and showed high clinical application value. MRI can show the condition of TMJ well, and is an important means of examination and evaluation of TMD [3, 4]. In this study, we retrospectively analyzed the MRI results of 684 patients with TMDs in our hospital, and evaluated its value in assisting the diagnosis of TMD.

## Methods

### Clinical data

The subjects were patients with TMD diagnosed clinically in the Department of Stomatology, Beijing Friendship Hospital affiliated to Capital Medical University from January 2017 to July 2018. All patients received a telephone return visit. The study was conducted in accordance with the Declaration of Helsinki (as revised in 2013). The study was conducted in accordance with the Declaration of Helsinki and approved by the Ethics Committee of Beijing Friendship Hospital Affiliated to Capital Medical University (NO. 2020-P2-209-02), and informed consent was taken from all the patients.

There were 684 patients with complete clinical and imaging data, including 166 males and 518 females, aged from 12 to 82 years, with an average age of 35.8 years. Inclusion criteria: (1) Chief complaint is bilateral joint area symptoms (including pain, popping and/or snapping noise during both opening and closing movement, limited opening); (2) Agree to be volunteer for the study; (3) MRI results are available. Exclusion criteria: (1) The patient may have a disease such as a tumor in the joint area (such as osteochondroma and synovial chondroma); (2) Suspected pain associated with jaw and/or salivary glands, unexplained headache; (3) MRI image quality is poor and difficult to evaluate.

Diagnostic criteria [5] were: (1) disc displacement with reduction: (a) clicking, popping and/or snapping noise during both opening and closing movement, detected with palpation during at least one of three repetitions of jaw opening and closing; (b) clicking, popping and/or snapping noise detected with palpation during at least one of three repetitions of opening or closing movements; (c) clicking, popping and/or snapping noise detected with palpation during at least one of three repetitions of right and left lateral, or protrusive movements. (2) Disc displacement with reduction with imtermittent locking: (a) Clicking, popping and/or snapping noise detected during both opening and closing movements, detected with palpation during at least one of three repetitions of jaw opening and closing; (b) clicking, popping and/or snapping noise detected with palpation during at least one of three repetitions of opening or closing movements; (c) clicking,popping and/or snapping noise detected with palpation during at least one of three repetitions of right and left lateral,or protrusive movements. (3) Disc displacement without reduction with limited opening: (a) Jaw locked so that the mouth would not open all the way; and limitation in jaw opening severe enough to limit jaw opening and interfere with ability to eat; (b) maximum assisted opening (passive stretch) movement including vertical incisal overlap < 40mm; (4) Disc displacement without reduction without limited opening: (a) jaw locked so that the mouth would not open all the way; and limitation in jaw opening severe enough to limit jaw opening and interfere with ability to eat; (b) maximum assisted opening (passive stretch) movement including vertical incisal overlap > 40mm; (5) myalgia: (a) confirmation of pain locations in the temproralis or masseter muscle; (b) report of familiar pain in the temporails or masseter muscles with at least one of the following provocation tests: palpation of the temporails or masseter muscles; or maxinmum unassisted or assisted opening movements.

### Examination method

Excite-II superconducting MR machine of GE Company was used for scanning, bilateral temporomandibular joint surface coils (DUAL coils) were used for transection, oblique sagittal and oblique coronal examination of opened and closed mouth. The oblique sagittal scan plane was perpendicular to the long axis of the condyle, enclosing the left and right sides of the condyle; the oblique coronal scan plane was parallel to the long axis of the condyle. MRI scanning sequences included: closed-mouth sagittal T1WI and PDWI, coronal T1WI and T2WI, opened-mouth sagittal TIWI and PDWI.

Two professional radiologists were selected for training according to diagnostic criteria, and the Kappa consistency test was performed on the diagnostic results of these two radiologists. After the Kappa value reached above 0.63, two radiologists were arranged to re-read the MRI images of 684 patients with TMDs to obtain the diagnostic results. At the same time, we retrospectively followed up with these 684 TMD patients and collected clinical data.

**Fig. 1:**
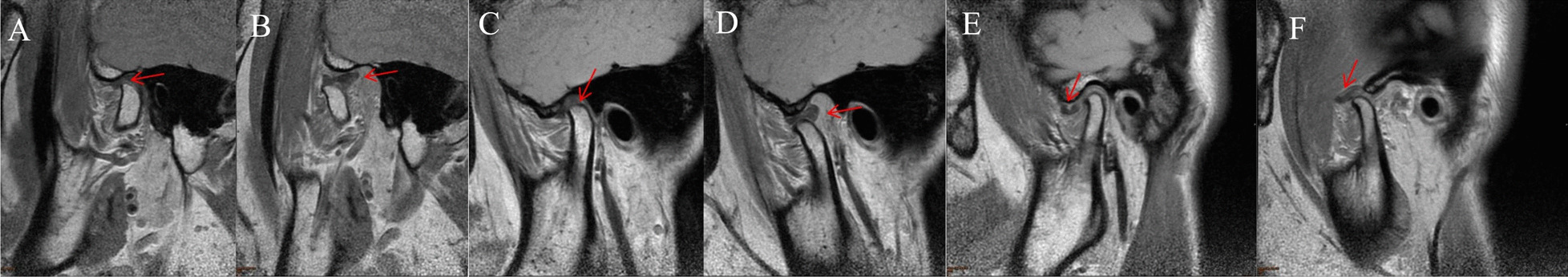
Normal intervertebral disc and its displacement. Normal disc-condyle relationship, the articular disc is located above the top of the condyle, as shown by the red arrow. The closed-mouth (**A**) and opened-mouth position (**B**) of normal intervertebral disc were presented. Disc displacement without reduction, the posterior zone of the articular disc lies in front of the condyle. The closed-mouth (**C**) and opened-mouth position (**D**) of disc displacement without reduction were presented. Disc displacement with reduction, the posterior zone of the articular disc lies in front of the condyle. The closed-mouth (**E**) and opened-mouth position (**F**) of disc displacement with reduction were presented

The diagnostic classification criteria for MRI images are as follows:

Disc displacement with/without reduction: There is a clear demarcation line (disc demarcation line) between the posterior zone of the articular disc and the bilateral plate area on TIWI images, the posterior zone of the articular disc is located at the top of the condyle. If the angle formed by the disc demarcation line and the 12-point vertical line of the condyle is within 10 degrees, it is a normal disc-condyle relationship; if the disc demarcation angle is more than 10 degrees forward, it is anterior disc displacement. In opened-mouth oblique sagittal position, if the disc-condyle relationship returns to normal, it is disc displacement with reduction. If the disc-condyle position cannot be restored, it is disc displacement without reduction, as shown in Fig. 1.

**Fig. 2:**
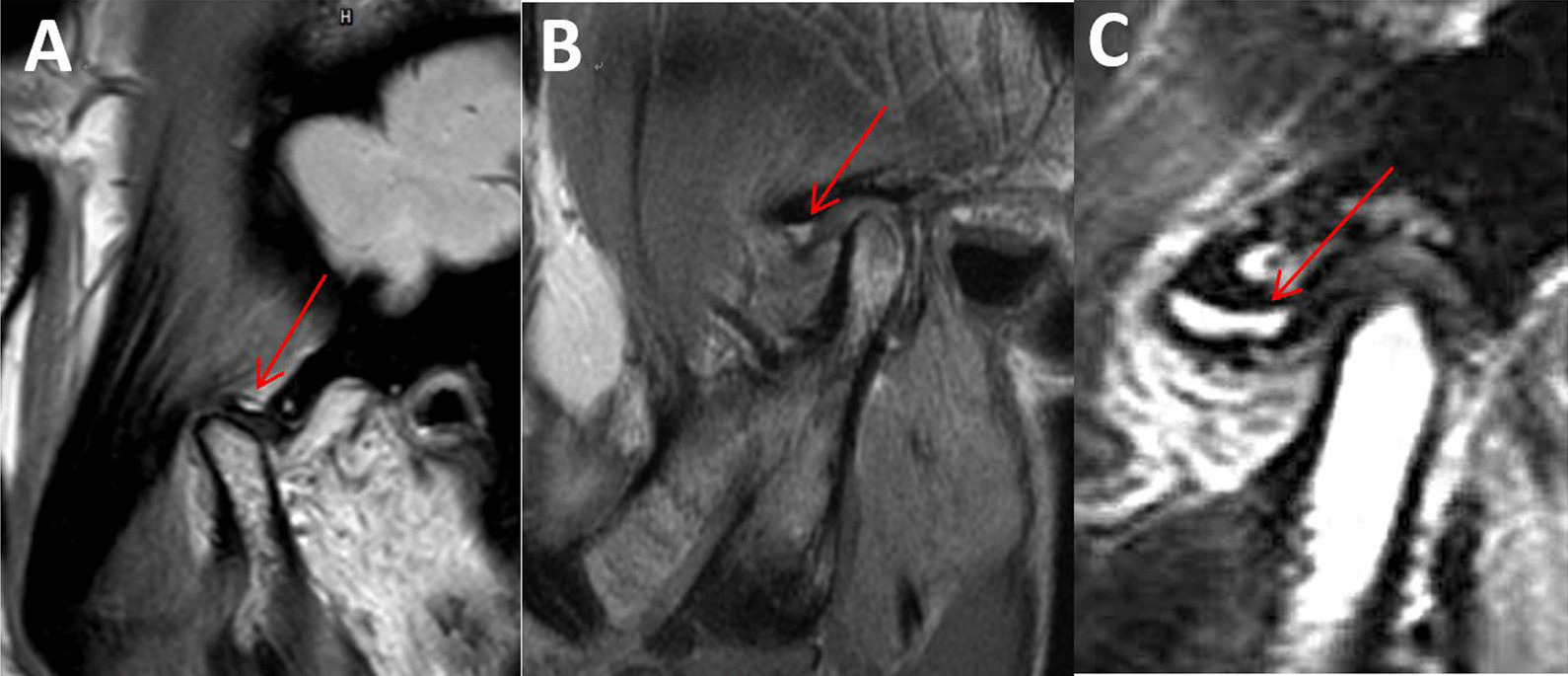
On T2WI images, joint effusion can be seen in the joint cavity. **A** Mild: punctate or linear high-density shadow can be seen in the upper joint cavity. **B** Moderate: between mild and severe. **C** Severe: patchy high-density shadow can be seen in the upper joint cavity

Joint cavity effusion: There is joint effusion in the joint cavity on the T2WI image. Mild: punctate or linear high-density shadow can be seen in the upper joint cavity. Severe: patchy high-density shadow can be seen in the upper joint cavity. Moderate: between mild and severe (Fig. 2).

**Fig. 3:**
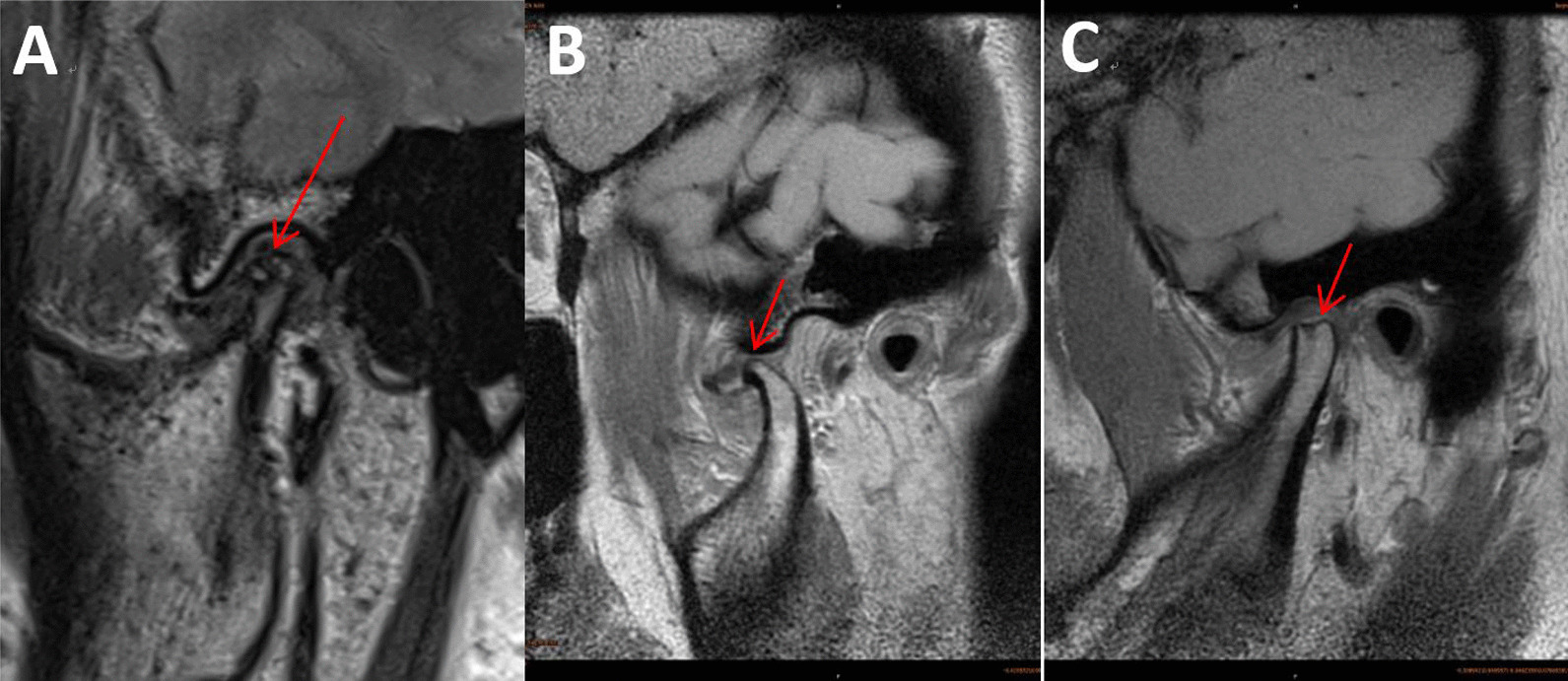
Sagittal MRI of TMJ. **A **Osteophytosis. **B** Erosion and sclerosis. **C** Mandibular condylar erosion and joint eminence

Osteoarthritis: The morphology of the condyle changes on MRI images, with osteophytes, erosion and sclerosis, and mandibular condylar erosion and joint eminence can be seen (Fig. 3).

### Statistical analysis

All the data were analyzed using SPSS 22.0 software in this study. Measurement data were expressed as mean ± standard deviation (mean ± SD), and the comparisons between groups were performed using the independent sample *t*-test. The categorical data were expressed as n (%), and their differences between the two groups were examined by the Pearson *χ*^*2*^ test. The differences in clinical variables between groups were tested using univariate analysis, and the variables were included in the binary multivariate logistic regression analysis to confirm whether they were independent risk factors. *P* < 0.05 was considered statistically significant.

## Results

### Clinical data statistics of 684 patients with TMD

There were 684 patients with TMD, and the male-to-female ratio was about 1:3. The average age of the patients was 35.8 years, and the age range was 12–82 years. All the diagnostic groups showed obvious stability, and the diagnostic results were as follows: 68 cases without bilateral abnormalities (23 males and 45 females); 88 cases with bilateral disc displacement without reduction (9 males and 79 females); 152 cases with bilateral reducible disc anterior displacement (43 males and 109 females); 104 cases (18 males and 86 females) with one side disc displacement with reduction and disc displacement without reduction. 94 cases (18 males and 76 females) had normal one side, 119 cases (35 males and 84 females) had disc displacement with reduction on one side, and 58 cases had joint cysts, osteoarthritis and other organic changes.

### Relationship between disc displacement with/without reduction and age

**Fig. 4:**
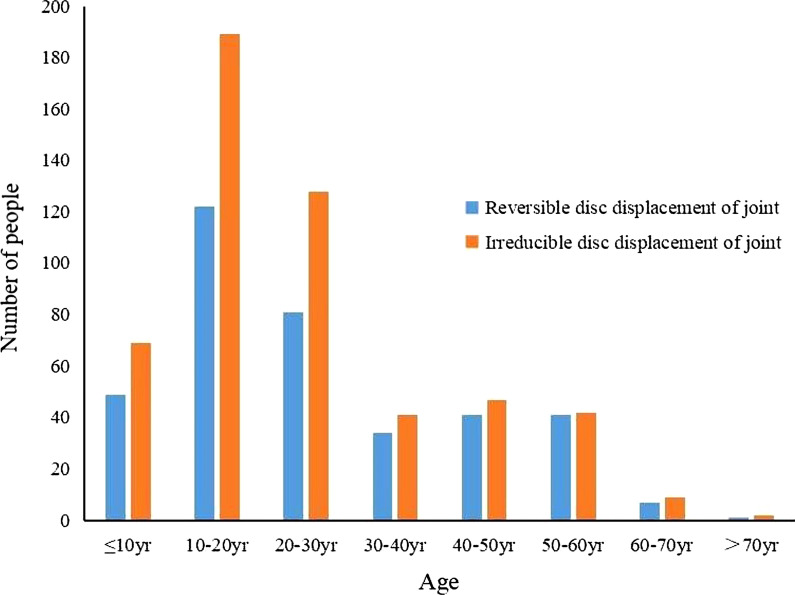
The relationship between MRI findings of TMJ and age

The relationship between MRI findings of TMJ and age is shown in Fig. 4. The largest proportion of these 684 patients in our hospital was 20 to 29 years old. Chi-square test showed that there was a significant difference in the number of male and female patients with at least one side of the disc displacement (*P* = 0.002). 22.6 % of the patients were male and 67.4 % were female.

### Distribution of age in patients with osteoarthritis

**Fig. 5:**
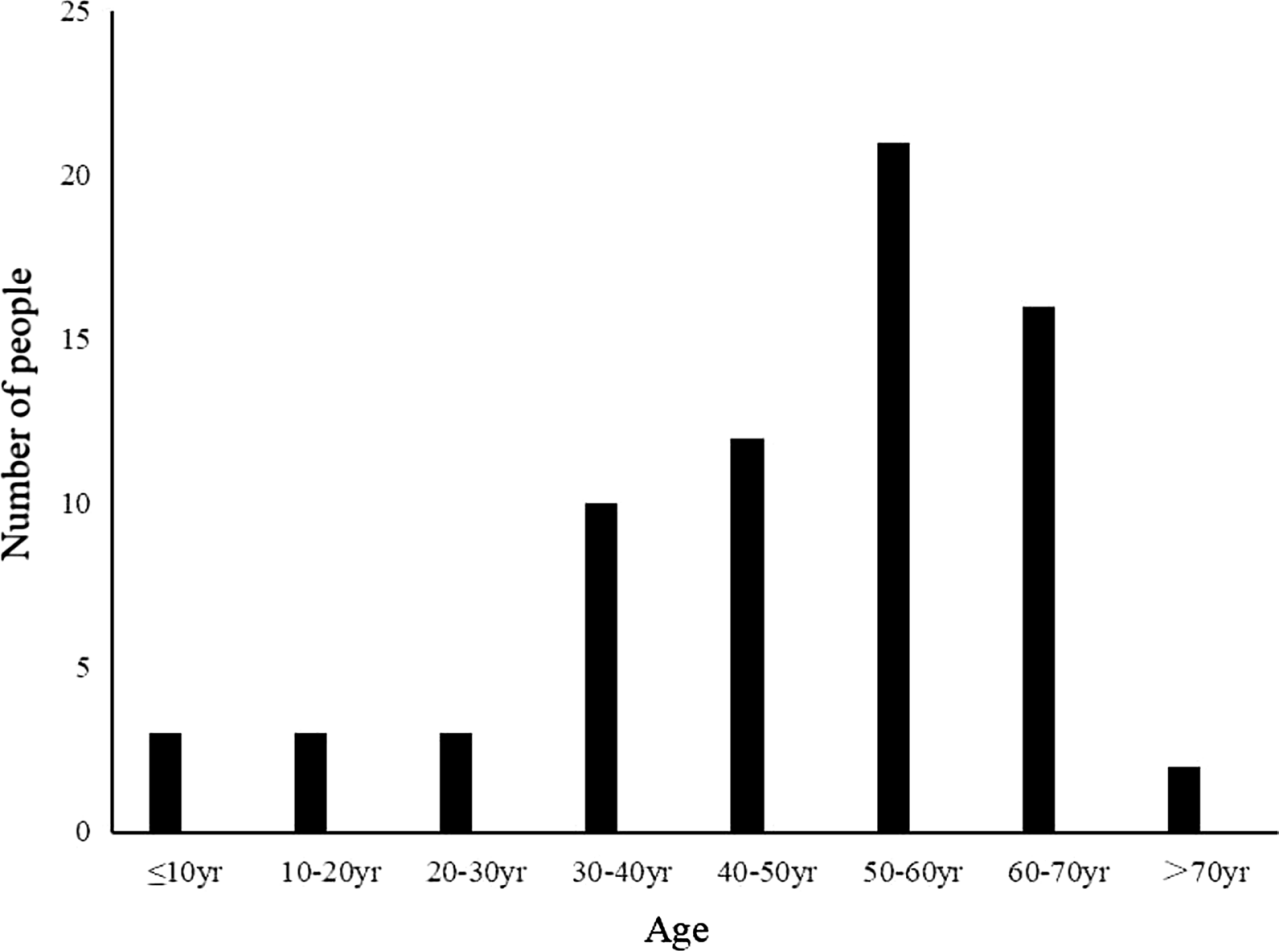
Age-related diagnosis of osteoarthritis by TMJ MRI

Among these 684 patients with TMD treated in our hospital, 82 patients were over 60 years old, of which 60 patients had MRI results showing osteoarthritis on at least one side (Fig. 5). So we further studied the MRI results of osteoarthritis in the elderly.

Among these 82 elderly patients older than 60 years, 52.4 % of them showed no signs of osteoarthritis on MRI images of the condyle, and 47.6 % of the patients showed signs of osteoarthritis on at least one side of the condyle, as shown in Table 1. There was no significant difference in the incidence of osteoarthritis between male and female (*P* = 1.000). The proportion of male and female patients diagnosed with osteoarthritis was 32 and 68 % respectively. To further explore the MRI features of TMJ disc displacement in the elderly patients, patients over 60 years old with anterior disc displacement were selected as the elderly group, and the remaining patients (< 60 years old) with anterior disc displacement were selected as the control group. Each side of the anterior disc displaced joints was taken as an independent study subject. In this study, 684 patients with 1368 single joints were included. Among them, 901 joints had disc displacement, including 101 in the elderly group and 800 in the control group. The results of the study are shown in Table 2.

**Table 1:** MRI findings of osteoarthritis over 60 years of age

Osteoarthritis	Number	Percentage (%)
Normal	43	52.4
Unilateral osteoarthritis	29	35.4
Bilateral osteoarthritis	10	12.2

**Table 2:** Analysis of MRI in patients with anterior disc displacement in the elderly group and control group

	ADD		Joint effusion		Osteoarthritis		Total
	RE (%)	IR (%)	Y	N	Y	N	
Elderly group	52 (51.4%)	49 (48.5%)	3 (3.0%)	98 (97.0%)	38 (37.6%)	63 (62.4%)	101 (100%)
Control group	475 (59.4%)	325(40.6%)	14 (1.8%)	786 (98.2%)	61 (7.7%)	739 (92.3%)	800 (100%)
Total	527 (58.5%)	374 (41.5%)	17 (1.9%)	884 (98.1%)	99 (11%)	802 (89.0%)	901 (100%)
*P*	0.135		0.425		< 0.001		

ADD, Anterior disc displacement; RE, reversible; IR, irreversible

The proportion of disc displacement with reduction was not significantly different between the elderly group (48.5 %) and the control group (40.6 %) (*P* = 0.135). The percentage of elderly patients with anterior disc displacement accompanied by joint effusion (3.0 %) was not significantly different from that of the control group (1.8 %) (*P* = 0.425). The proportion of elderly patients with osteoarthritis in anterior disc displacement was significantly higher than that in the control group (37.6 % vs. 7.7 %, *P* < 0.001).

We then performed logistic regression analysis on the MRI characteristics of the elderly patients with anterior disk displacement to identify key risk factors, and the results are shown in Table 3. The results confirm that osteoarthritis is an important risk factor for elderly patients with anterior disc displacement.

**Table 3:** Logistic regression analysis on the MRI characteristics of the elderly patients

MRI Diagnosis	OR	95 % CI	*P*
Anterior disc displacement	0.855	0.55–1.327	0.484
Joint effusion	2.265	0.591–8.687	2.265
Osteoarthritis	0.129	0.078–0.212	< 0.001

## Discussion

TMD is a common disease caused by multiple factors. It is common in young and middle-aged people, mainly manifested by joint pain, clicking, limited opening and other functional disorders. Effective treatment of TMDs depends on the correct diagnosis of the disease. In the past, oral surface tomography and CT were commonly used as auxiliary examinations. However, these two methods have poor imaging of the soft tissue lesions of the temporomandibular joint, such as the location, shape and perforation of the articular disc. In recent years, the technology) of MRI for the examination of TMD has become increasingly mature, and has gradually become the preferred method for the diagnosis of TMD.

In this study, a total of 696 patients with TMD had MRI images. After excluding patients with poor image quality and incomplete clinical data, the remaining 684 patients were retrospectively analyzed. In previous studies on TMD, the male-to-female ratio ranged from 1:1.75 to 1:3 in most studies [6, 7]. The male-to-female ratio in this study was about 1:3, which is consistent with this observation. There is a view that female patients are significantly higher than male patients because of increased demand for treatment in female patients, rather than because women are more susceptible to TMD [8]. Another view is that estradiol-female sex hormones can induce proinflammatory cytokines and aggravate TMJ inflammation [9]. Previous studies have deeply explored the causes of male-female differences in the incidence of TMDs, and analyzed factors such as endocrine, physical, psychosocial and behavioral factors, but these studies have not yet reached definite conclusions.

In this study, patients were mainly 20–30 years old, accounting for 57.1 %. In most studies, like Kwang’s, the male-to-female ratio of the subjects was about 1:3 [10], which was the same as 65.8 % of the patients with anterior disc displacement in this study (27.3 % with disc displacement without reduction and 38.5 % with disc displacement with reduction). In this study, the age at which disc displacement with/without reduction occurred was mostly 20–30 years old.

Osteoarthritis is a disease formerly known as an organic change of the joint. Degenerative changes in articular bone, cartilage, and discs can be detected by MRI. Although CT has higher specificity and sensitivity than MRI in the diagnosis of bone changes, Liu et al. found the accuracy of MRI in determining the location, morphology and evaluation of intervertebral discs [11]. The diagnosis of osteoarthritis should be based on the evaluation of MRI images in combination with clinical examination. It is generally believed that the most common pathological manifestation of the condylar process is articular surface degeneration [12]. Osteoarthritis and internal disorders coexist in only one-third of cases, which means that the view that subclinical osteoarthritis leads to the pathological tissue response to internal disorders should be reexamined [13]. Temporomandibular joint-related pain can be predicted by temporomandibular joint MRI [14–17]. In this study, we used MRI to assess bone changes in the TMJ and studied bone changes caused by disc position. In addition to the above symptoms, continuous fricative or crushing sounds can be heard during joint movement. The self-conscious symptoms of this kind of disease are not obvious in the stable stage, and there is no obvious dysfunction. Some damaged bones can be repaired after treatment or several months of recuperation. Some patients are accompanied by a certain degree of synovitis or capsulitis, with obvious symptoms, pain in opening and chewing, limited opening, and sometimes recurrent attacks, which can be called osteoarthritis. The morphology of the condyle changes on MRI images, including osteophytosis, erosion and sclerosis, and protrusion of the mandibular condylar joint.

Although temporomandibular osteoarthritis is a common basic type of disease, there is a lack of standardized clinical data and imaging information on osteoarthritis in the elderly. In this study, a total of 79 patients with TMJ osteoarthritis, accounting for about 11.5 % of the total, were mainly concentrated in people aged 60–70 years, which may be that the prevalence of osteoarthritis increases with age. The decline in the prevalence of osteoarthritis in the 70–80 years may be due, on the one hand, to a relative reduction in the number of elderly people, and on the other hand, to a reduced demand for treatment for TMD in elderly patients. Further analysis showed that the prevalence of osteoarthritis was 73.2 % in patients older than 60 years, although the subjective symptoms of osteoarthritis were rarely found clinically. This result confirms the view put forward by Bagge [18] that moderate and severe osteoarthritis may also occur without any conscious symptoms. Bernhardt [19] performed clinical and MRI examinations in male and female of different age groups to assess the incidence of degenerative changes in the TMJ and found that the prevalence was 25 %. Furthermore, these authors found a joint pain rate of 12.7 %, which seems to contradict the results of this study. However, in Bernhardt’s study, subjects were mostly 20–49 years old, so the lower prevalence of osteoarthritis is easy to explain. Some studies have shown that the subjective symptoms of TMJ osteoarthritis decrease with age, while the objective symptoms increase with age [20]. We investigated whether anterior disc displacement of the TMJ is associated with osteoarthritis. In this study, we believe that there is no correlation between the two and they develop independently, which seems to contradict the mainstream view. Gil C et al. concluded that the development of anterior disc displacement of TMJ was not associated with osteoarthritis if TMJ was considered as a whole, but specific subclassifications in osteoarthritis were associated with anterior disc displacement [21]. In future studies, we will further explore which specific type of osteoarthritis is associated with anterior disc displacement.The MRI examination of the temporomandibular joint is affected by the age of the population, the frequency of the examination, the tolerance of the patient, the follow-up and other factors. It can help to conduct an early intervention for TMD. At the same time, the development of MRI technology has also significantly increased the detection rate of TMD. However, the diseases in a large proportion of patients are self-limiting and can return to normal without external intervention. Therefore, this study found that in the diagnosis of TMD, there are varying degrees of overdiagnosis, which has a negative impact on patients and families. The main factor affecting overdiagnosis is the self-limiting nature of TMD. Therefore, in order to avoid over-diagnosis as much as possible and improve the accuracy of clinical diagnosis, it is particularly important for clinicians to grasp the criteria for correct diagnosis of TMD.

## Conclusions

Bilateral temporomandibular joint MRI can clearly show TMJ changes and can be used to diagnose TMD; there are significantly more female with TMD than male and osteoarthritis has a significant correlation with age.

## Data Availability

The datasets in this study are available from the corresponding author on reasonable request.

## Abbreviations

MRI: Magnetic Resonance Imaging
TMD: Distinguishing temporomandibular disorders
TMJ: Temporomandibular joint
